# Exploiting single-cell expression to characterize co-expression replicability

**DOI:** 10.1186/s13059-016-0964-6

**Published:** 2016-05-06

**Authors:** Megan Crow, Anirban Paul, Sara Ballouz, Z. Josh Huang, Jesse Gillis

**Affiliations:** Cold Spring Harbor Laboratory, One Bungtown Road, Cold Spring, Harbor, NY 11724 USA

**Keywords:** Co-expression, Meta-analysis, RNA-seq, Single cell, Network, Normalization, Brain, Interneuron, Autism

## Abstract

**Background:**

Co-expression networks have been a useful tool for functional genomics, providing important clues about the cellular and biochemical mechanisms that are active in normal and disease processes. However, co-expression analysis is often treated as a black box with results being hard to trace to their basis in the data. Here, we use both published and novel single-cell RNA sequencing (RNA-seq) data to understand fundamental drivers of gene-gene connectivity and replicability in co-expression networks.

**Results:**

We perform the first major analysis of single-cell co-expression, sampling from 31 individual studies. Using neighbor voting in cross-validation, we find that single-cell network connectivity is less likely to overlap with known functions than co-expression derived from bulk data, with functional variation within cell types strongly resembling that also occurring across cell types. To identify features and analysis practices that contribute to this connectivity, we perform our own single-cell RNA-seq experiment of 126 cortical interneurons in an experimental design targeted to co-expression. By assessing network replicability, semantic similarity and overall functional connectivity, we identify technical factors influencing co-expression and suggest how they can be controlled for. Many of the technical effects we identify are expression-level dependent, making expression level itself highly predictive of network topology. We show this occurs generally through re-analysis of the BrainSpan RNA-seq data.

**Conclusions:**

Technical properties of single-cell RNA-seq data create confounds in co-expression networks which can be identified and explicitly controlled for in any supervised analysis. This is useful both in improving co-expression performance and in characterizing single-cell data in generally applicable terms, permitting cross-laboratory comparison within a common framework.

**Electronic supplementary material:**

The online version of this article (doi:10.1186/s13059-016-0964-6) contains supplementary material, which is available to authorized users.

## Background

**Fig. 1:**
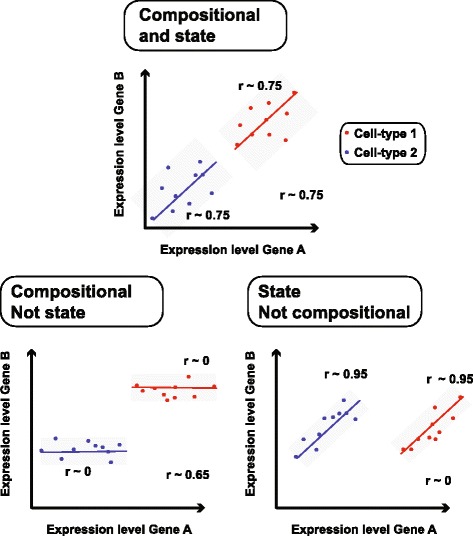
What lies beneath: co-expression can reflect different combinations of cell-state or compositional variation. Each *panel* shows a different scenario in which cell state and composition affect the expression of two genes (A and B), yielding different types of co-expression. Two cell types are colored in *red* and *blue*. In the *top panel*, both cell types have state-dependent variation that causes co-expression within each (r ~ 0.75). In addition, there is co-expression due to compositional variation (r ~ 0.75). In the *bottom left panel* only compositional variation is apparent (r ~ 0.65), there is no relationship between gene A and gene B within the cell types (r ~ 0). This is the opposite in the *bottom right panel*. Here, there is only variation within the cell types (r ~ 0.95) but no compositional effect across cell types (r ~ 0). The exact value the compositional correlations take would vary in real data since combinations of the underlying cell types would fill in intermediate points, but the three cases would still occur as described; other possibilities due to noise or other complex scenarios (e.g. Yule-Simpson effect) are also possible

Biology has increasingly looked to relationships between genes to explain phenotypic variability. One way to determine these functional groupings is from transcriptional data; genes with similar expression patterns are thought to be involved in the same cellular pathway or function [1]. Networks derived from expression data have become an important resource in the interpretation of gene function [2] and disease [3]. Co-expression networks are built from an assessment of similarity, often correlation, between gene pairs across sources of variation (see Box 1 for more detail). For bulk RNA sequencing (RNA-seq) and microarray data, the sources of variation are manifold, and pinpointing driving factors has been challenging. For example, co-expression signals may be interpreted as reflecting compositional differences, such as varying proportions of underlying cell types within a tissue, or cell-state differences, like the circadian rhythm, or some combination of both, with data quality and technical variation further complicating interpretation (see Fig. 1).

Single-cell RNA-sequencing (scRNA-seq) data provide the opportunity to gain insight into expression heterogeneity at finer resolution. scRNA-seq has now been applied to many human and mouse tissue types at multiple stages of development, including the lung, spleen, brain, retina, embryonic stem cells, and lumbar dorsal root ganglia, among others [4–11]. Because the primary aim of many scRNA-seq studies is to determine novel, transcriptionally defined cell types, most computational work in this area has focused on unsupervised clustering and differential expression, techniques that are affected by the technical variability and low data coverage inherent to scRNA-seq (for review see [12]). Co-expression of scRNA-seq data remains relatively uncharted territory (although see [6, 10, 13–19]). The increased prevalence of single-cell data makes it possible to consider its co-expression properties in aggregate, where functional signals are most robust [20].

Here we have attempted the first major analysis of single-cell co-expression, including a meta-analysis of scRNA-seq expression, sampling from 31 individual studies comprising 163 individual cell types (Table 1). By comparing networks made from individual cell types to networks containing all of the cell types assayed within an experiment, we can assess the effects of cell-state and compositional variation on functional connectivity (where “functional” refers to known overlaps with gene sets defined to have a common function by the Gene Ontology [GO]). In addition, we compared single-cell data to 239 bulk RNA-seq experiments as an external control (Additional file 1: Table S1). From these data, we found that single-cell network connectivity is significantly predictive of function, particularly in aggregate, but is less likely to overlap with known functions than co-expression derived from bulk data. Most interestingly, assessing single-cell data in which cell type was held constant in each network (i.e. excluding compositional co-expression) showed little decrease in performance on this task, suggesting that gene sets varying from cell to cell within a cell type are similar to those that vary from cell type to cell type.

**Table 1:** Single-cell RNA-seq expression studies used for meta-analysis, sorted by GEO ID (GEO ID = Gene Expression Omnibus Identifier). Experiments were defined by unique GEO ID

First author	Year	Journal	GEO ID	Samples	Cell types
Deng	2014	Science	GSE45719	266	9
Streets	2014	PNAS	GSE47835	56	1
Treutlein	2014	Nature	GSE52583	184	6
Jaitin	2014	Science	GSE54006	12	1
Kim	2015	Cell Stem Cell	GSE55291	56	3
Biase	2014	Genome Research	GSE57249	40	1
Kowalczyk	2015	Genome Research	GSE59114	1933	12
Brunskill	2014	Development	GSE59127	86	1
Brunskill	2014	Development	GSE59129	49	1
Brunskill	2014	Development	GSE59130	57	1
Usoskin	2015	Nature Neuroscience	GSE59739	638	5
Sansom	2014	Genome Research	GSE60297	174	1
Zeisel	2015	Science	GSE60361	2975	7
Kumar	2014	Nature	GSE60749	415	4
Velten	2015	Molecular Systems Biology	GSE60768	100	3
Moignard	2015	Nature Biotechnology	GSE61470	15	1
Grun	2015	Nature	GSE62270	1154	12
Macosko	2015	Cell	GSE63472	8120	38
Li	2015	Cell Research	GSE63576	204	1
Lindeman	2015	Current Biology	GSE64960	67	1
Klein	2015	Cell	GSE65525	8649	6
Chen	2015	Developmental Cell	GSE66202	91	3
Du	2015	Thorax	GSE69761	148	1
Fuzik	2015	Nature Biotechnology	GSE70844	76	1
Shin	2015	Cell Stem Cell	GSE71485	167	2
Tasic	2015	Nature Neuroscience	GSE71585	1740	8
Burns	2015	Nature Communications	GSE71982	278	13
Gaublomme	2015	Cell	GSE74833	722	13
Kimmerling	2015	Nature Communications	GSE74923	178	2
Hanchate	2015	Science	GSE75413	84	4
Fan	2016	Nature Methods	GSE76005	65	1

To complement this analysis, we performed our own technically controlled scRNA-seq experiment using genetically targeted interneuron classes to further interrogate data features and analysis practices that contribute to functional connectivity in co-expression networks. Chandelier cells and parvalbumin-positive fast-spiking basket cells were prepared in a series of batches of 16 cells to generate co-expression networks for each [21]. This allowed us to take the same meta-analytic approach we took to cross-laboratory comparison to characterization of technical properties within our data by performing a meta-analysis across batches. We focused on the principal source of variation reported on in MAQC-III, library preparation, which was done independently for each batch [22]. In addition, because normalization plays a critical role in technical assessment, we used varietal tags [23] (similar to unique molecular identifiers [UMI]) to measure discrete expression values. We then assessed a number of approaches for parsing the data, attempting to sample from fundamental statistical methods whose results are highly likely to generalize to new approaches and whose output is readily interpretable and robust (i.e. not prone to overfitting).

This strategy proved successful as we were able to delineate straightforward heuristics to inform the design and interpretation of scRNA-seq co-expression analysis. In short, we found that the use of raw or batch-corrected UMI data and post-co-expression network standardization provided the highest degree of network replicability, semantic similarity among top connections, and overall functional connectivity. However, we found that gene expression levels within these networks were highly predictive of node degree and functional connectivity, which led us to propose control experiments, like the use of expression-matched control gene sets, to assess performance specificity. This finding further allowed us to tunnel into the known dependency of network performance on node degree [24], which we reproduced in this analysis of bulk and single-cell data. Based on this, we made the prediction that previous claims about age-related co-expression specificity of autism candidate genes using the BrainSpan RNA-seq data [25] may benefit from control for the possibility of differential expression across the same data. Indeed, we found evidence that variation in autism gene connectivity was predicted by expression level, with the highest performance and expression in pre-natal networks, but the highest dependency on this potential confound within post-natal networks.

Our results have a number of direct implications for single-cell analysis and co-expression more generally:

- Where co-expression can be explained by simpler effects, it is important to do so. Expression level dependence should be a default control for all co-expression analyses.
- Normalization of data is not independent of the use to which that data will be put. In particular, sample normalization which is helpful for differential expression may be damaging for co-expression. As in expression-level dependence, single-cell data offer particular clarity on the role of these effects.
- Compositional co-expression likely overlaps with state co-expression. Whether this is because our knowledge of gene function is not yet cell type-specific or because co-expressed genes form similar functional units across cells or cell types is a question of importance for future biological research.

While our analysis suggests technical concerns will be critical to the interpretation of scRNA-seq co-expression for some time, careful experimental design and analysis choices, such as the inclusion of replicates and testing for technical confounds, can overcome these issues and open new avenues for biological research.

## Results and discussion

### Meta-analysis of scRNA-seq co-expression

Co-expression networks have been used to provide important clues about the cellular and biochemical mechanisms that may be active in normal and disease processes. An outstanding question in the characterization of co-expression is the relative importance of variable sample composition in real terms (e.g. the proportion of neurons vs. glia in a brain sample) versus variability in cell state (e.g. the engagement of different molecular players throughout the phases of the cell cycle). The increasing availability of scRNA-seq data allows us to answer this question by comparing networks built from specified cell types, which should have only state-dependent co-variance, to networks built from ensembles of different cell types from the same experiment (Fig. 1). These pseudo-composite networks would have similar technical properties to the individual cell-type networks but would contain compositional variation in addition to state variation. Comparing these in meta-analysis across many experiments that use different biochemical and informatics protocols allows us to draw conclusions that are likely to generalize rather than being specific to any one technology or analysis practice.

Using a neighbor-voting algorithm in cross-validation we assessed the connectivity of a representative subset of GO functions (GO slim) in all networks. In essence, the algorithm predicts a gene will have a given function based on the proportion of its connectivity to genes that already have that function (i.e. the sum of the gene’s edge weights within the function divided by the gene’s node degree). This can then be assessed for correctness by holding back some functional labels, as is conventional in cross-validation. A network’s performance is the average score for each GO function and is reported as the area under the receiver operating characteristic curve (AUROC). The intuitive interpretation of the AUROC score is the probability that we would be right about classifying genes as belonging to a particular GO function or not. Our lab has done extensive testing and benchmarking of this algorithm against both more sophisticated machine learning methods, and using varying methods for network generation (e.g. partial correlation, mutual information, etc.), and found that results were robust [21, 26]. This gives us confidence that the approach is likely to generalize. Results from our single-cell analysis are discussed in more detail in the following sections.

#### Single-cell co-expression exhibits lower functional connectivity than that derived from bulk data

**Fig. 2:**
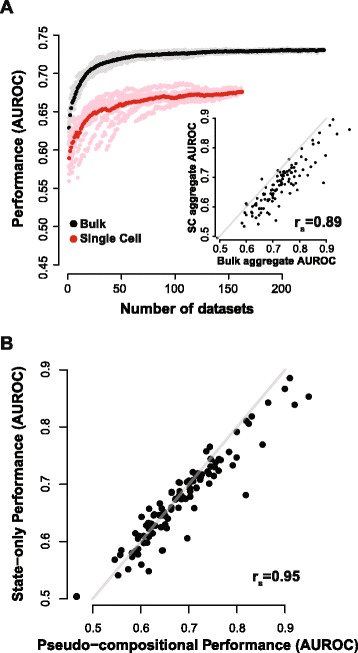
scRNA-seq networks have lower functional connectivity than bulk RNA-seq networks and state-driven performance is highly correlated with compositional performance. **a** Individual bulk and scRNA-seq networks were aggregated ten times in random order by averaging rank co-expression values, then re-ranking the resulting network. Receiver operating characteristic (ROC) scores for GO slim categories were calculated as each additional network was added. Individual runs are shown in *gray* (bulk) and *pink* (sc), and means are shown in *black* and *red*. Performance rises with aggregation for both bulk and sc networks, but sc networks have much lower baseline functional connectivity. *Inset:* Aggregate single-cell network (163 cell types) GO slim AUROCs are plotted against those of the aggregate bulk network (163 experiments). Performance is strongly correlated (r = 0.89) but is consistently lower in the single-cell aggregate. **b** Aggregate cell-type network GO slim AUROCs are plotted against those of the pseudo-compositional aggregate network. Performance is strongly correlated

scRNA-seq co-expression network performance has not previously been assessed, so to benchmark functional connectivity of single-cell networks, we first compared them to bulk RNA-seq co-expression networks where there is a prior expectation that networks built from 15 or more samples should show some non-random performance in predicting GO functions (i.e. they should have AUROCs >0.5, see [21] for more detail). We parsed data from 31 individual scRNA-seq studies into 163 cell types comprising 28,799 samples in total and 239 bulk RNA-seq experiments of similar sample sizes (Table 1 and Additional file 1: Table S1, and details in “Methods”). Signed, weighted networks were built for each cell type and for each bulk RNA-seq experiment by taking the Spearman correlation of all genes, then rank standardizing correlations between 0 and 1, which is a method to reduce the impact of experiment-specific factors such as outlier samples which could alter the correlation distribution between gene pairs substantially, but would have much less impact on their relative ranking. This produces networks in which each gene has a degree of connectivity with each other gene (i.e. a fully connected network); the higher the correlation between a gene pair, the higher the weight on their connection. Notably, individual single-cell networks had lower performance than bulk networks (Fig. 2a, mean sc AUROC = 0.56 +/– 0.002 SEM (used throughout), mean bulk AUROC = 0.60 +/– 0.002, *p* <2^–16^ Wilcoxon rank sum test, n = 163, = 239), indicating that the greater homogeneity of the single-cell data does not necessarily improve functional precision of co-expression networks.

Some of the power of meta-analysis comes from the ability to summarize data from multiple sources using a common analytic framework. In our case, this takes the form of network aggregation, where individual networks are combined to form an aggregate network [20, 21, 27]. We add networks one by one, permuting the order of combination through multiple runs, which allows us to measure the dependence of aggregate network performance, and variation in performance, on the number of experiments. For both bulk and single-cell networks, aggregation was repeated ten times and GO slim performance was assessed after each additional network was added (Fig. 2a); comparisons between bulk and single-cell aggregates were made using 163 datasets in each. As expected, aggregation improves performance for bulk networks (mean individual network AUROC = 0.60 +/– 0.001, aggregate AUROC = 0.73). Interestingly, aggregation also improves performance for single-cell networks, which indicates that there is replicable co-expression across diverse cell types (mean individual network AUROC = 0.56 +/– 0.002, aggregate AUROC = 0.68). AUROC scores are consistently lower in the single-cell aggregate than in the bulk aggregate, though performance across GO slim categories is well correlated (r_s_ = 0.89) (inset Fig. 2a). The use of another publicly available gene function prediction algorithm, GeneMANIA [28], yielded similar results (bulk aggregate AUROC = 0.70, sc aggregate AUROC = 0.61, GO slim performance correlation r_s_ = 0.74), indicating that this is a general property of the data, rather than being specific to our network analysis algorithm (Additional file 2: Figure S1).

Although single-cell networks have lower performance than bulk networks, their variation in performance is similar, with some exceptions (Additional file 2: Figure S1). We also find that variation is positively correlated with performance (Additional file 2: Figure S1). One possibility to account for this reduced performance is that single-cell co-expression aligns less with known biology due to some greater specificity; alternatively, it could reflect technical 1artefacts within single-cell data, such as batch effects or incomplete transcriptome coverage. We explore both possibilities in our own experiments below (see “Assessing practices affecting co-expression replicability and functional connectivity”).

#### Compositional variation does not add functional signal on top of that due to cell-state variation

The striking overlap between single-cell and bulk data in overall functional performance trends suggest a potential overlap between cell-state and compositional co-expression, the latter being likelier to occur in bulk data. To unravel these potential influences in driving functional connectivity within co-expression, we went on to generate pseudo-compositional scRNA-seq networks. These were built by taking Spearman correlations of all genes and all samples within an individual scRNA-seq study (including across cell types), then rank standardizing correlations between 0 and 1. Next, pseudo-compositional networks were aggregated and GO slim performance was compared to that of the cell-type aggregate. Interestingly, cell-type networks performed almost identically to pseudo-compositional networks (Fig. 2b), suggesting that compositional variation does not provide excess functional signal to cell-state variation (e.g. as in the top panel of the schematic, Fig. 1). This is further underscored by the lack of excess variation when comparing the standard deviation in performance across cell-type and pseudo-compositional networks; cell-type data are not visibly providing outlier functionality for networks from specific cell types (Additional file 2: Figure S1).

In sum, we find that single-cell networks have lower functional connectivity than bulk RNA-seq networks, likely due to the same technical issues that have been discussed in other contexts [29–33] and which are inherent to current scRNA-seq protocols (e.g. incomplete transcriptome coverage). In addition, it appears that variation in cell state alone is sufficient to produce functional signal within scRNA-seq networks and likely overlaps with variation across cell types. Because many single-cell studies are using their data to investigate cell state, our finding that known co-expression remains robust within data of constrained variation (e.g. single cell type) suggests that this may be a useful means of benchmarking the impact of technical effects. That is, we expect a known co-expression signal even in data that samples from previously unseen sources of variability and so we can assess methodological impacts even in wholly novel data. In the following section, we apply this approach to determine data features and analysis practices that contribute to functional connectivity in co-expression networks.

### Assessing practices affecting co-expression replicability and functional connectivity

Our meta-analytic results provided a functional connectivity benchmark for single-cell co-expression. Although co-expression replicated across networks, individual network performance was moderately variable (standard deviation in GO slim performance = 0.022, ~1/3 of AUROC above the null) and was not obviously predicted by sample size (r_s_ = 0.11), with the exception that very large experiments (>1000 samples) tended to have lower performance (mean AUROC of small experiments = 0.56 +/– 0.0001, mean AUROC large experiments = 0.5 +/– 0.02). Many technical and biological features vary across datasets, so to investigate the factors that might contribute to this variation with greater precision, we performed our own technically controlled scRNA-seq experiment. We profiled genetically targeted Chandelier cells (ChC) and parvalbumin-positive fast-spiking basket cells (Pv), two GABAergic interneuron types that are known to show some overlap and heterogeneity [34], making their characterization non-trivial but also a real use-case within randomly sampled data. Samples were prepared in known batches of 16 cells and we took advantage of varietal tag technology (similar to UMI) to measure discrete expression values [23] (sample details can be found in Additional file 3: Table S2). This experimental design allowed us to take a similar meta-analytic approach to that used in the previous section, but in this case treating each batch, rather than each cell type, as a replicate experiment. We aimed to test the impact of standardization, expression level, and drop-outs as these are the main features likely to have an impact, based on previous expression quality control [22] and single-cell analysis [29, 30, 35]. To do this we generated co-expression networks for each batch after varying the input data as specified (see Table 2), made aggregates for each input variant, then compared them to both known information (GO functions), described in “Characterization with respect to previously known function,” and for topological properties, described in “Characterization with respect to topology.” All assessments are summarized in Table 3.

**Table 2:** Network variations used for technical assessment. NB: for all networks undefined correlations are set to 0

Network	Method	Property tested
UMI	• Spearman correlation of UMI data to make a network for each batch • Batch networks are rank standardized then aggregated	Do UMI expression estimates produce functional co-expression?
CPM	• Spearman correlation of CPM normalized data to make a network for each batch • Batch networks are rank standardized then aggregated	What types of artifacts can sample standardization introduce?
Batch-affected	• Spearman correlation across all samples using UMI data • Rank standardization	What impact does co-variation across batches have?
Binary expression	• All non-zero values are set to 1 • Spearman correlation to make a network for each batch • Batch networks are rank standardized then aggregated	How informative is gene representation?
Combat	• UMI data is log2 transformed then Combat is run for each celltype (ChC and Pv) • Spearman correlation to make a network for each cell type • Aggregate is made from the addition of rank-standardized ChC and Pv networks	Do methods for removing batch effects alter co-expression?
Removal of unwanted variation (RUV)	• UMI data is log2 transformed then RUV is run for each cell type (ChC and Pv) using ERCC spike-ins as control genes • Spearman correlation to make a network for each cell type • Aggregate is made from the addition of rank-standardized ChC and Pv networks	What are the combined influence of batch correction and ERCC-based normalization?
UMI excluding zeroes	• All zeroes are set to NA • Networks are made for each batch using pairwise Spearman correlation • Batch networks are rank standardized then aggregated	How does removing zeroes alter network topology and performance?

**Table 3:** Methods used to assess co-expression networks

Assessment	Property tested	Method and interpretation
Co-expression performance	Functional connectivity of each GO group	• Neighbor voting for GO functions, threefold cross-validation
		• AUROC >0.5 means the genes within the GO group have greater connectivity than may be expected by chance
Within-network semantic similarity (thresholded)	Functional connectivity of the network	• Number of GO functions in common among top 1 % of network connections
		• A higher value indicates greater functional similarity among top connections
Between-network similarity (thresholded)	Topology	• Jaccard index (intersect/union) over top 1 % connections
		• Values closer to 1 imply greater similarity between networks
Co-expression performance after aggregation	Replicability of functional connectivity	• Neighbor voting for GO functions as datasets are added together to form aggregate networks
		• Improvement with aggregation suggests specific replicability of functional connections
Aggregate connectivity distributions	Replicability of topology	• Rank standardized networks (with uniform distributions) are added together to form an aggregate network, then the standard deviation across all co-expression values is measured
		• Higher variance indicates greater replicability of topology among networks

#### Characterization with respect to previously known function

**Fig. 3:**
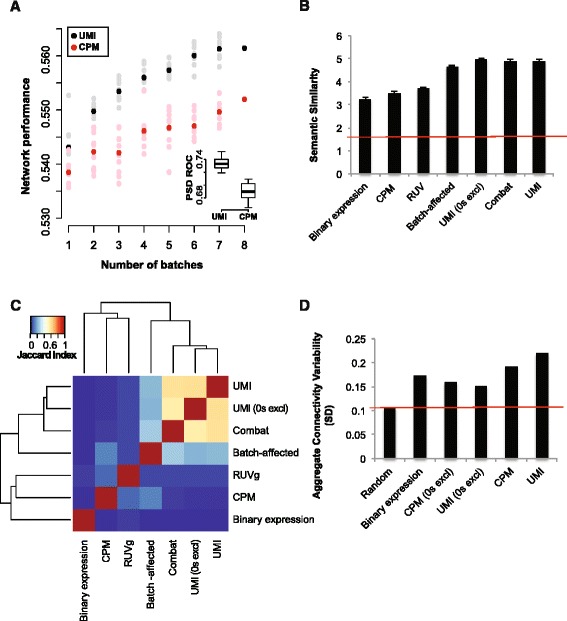
Comparative network analysis shows higher functional connectivity, semantic similarity, and convergent co-expression of UMI-based aggregates. **a** For each batch network, functional connectivity was benchmarked against 108 GO slim categories then networks were randomly selected and aggregated ten times. Networks built from raw UMI data are shown in *black* and count-per-million (CPM) standardized data are shown in *red*. As in our meta-analysis of single-cell networks, performance rises with aggregation, indicating an overlap in functional signal among networks. CPM networks have significantly lower functional connectivity than UMI networks (mean UMI = 0.54 +/– 0.002, mean CPM + 0.54 +/– 0.001, *p* <0.05 Wilcoxon rank sum, n = 8). *Inset: Boxplot* of synaptic gene performance for UMI and CPM networks. Though mean GO slim performance is modest, connectivity of this functionally relevant gene set is high (mean UMI = 0.73 +/– 0.004, mean CPM = 0.69 +/– 0005). **b** Semantic similarity of top 1 % network connections assessed by the number of shared GO functions. The *red line* indicates mean semantic similarity of all genes. Lower semantic similarity is observed for CPM, removal of unwanted variation (RUV), and binary expression aggregates compared to UMI-based networks. **c** *Plot* shows pairwise comparisons of top 1 % network connections based on the Jaccard index. UMI-based networks are more similar to one another than to CPM, RUV, and binary expression networks. **d** Standard deviation of aggregate co-expression values, *red line* marks the amount of variance expected by chance. All aggregates are more variable than random, indicating the presence of replicable co-expression among individual networks

For differential expression, it is standard to perform sample-level standardization to ensure that technical factors, like sequencing depth, do not obscure class differences. UMI data are typically standardized by dividing the count of the number of molecules by the total count per sample, then multiplying by a large number (sometimes called TPM or CPM for “counts-per-million” normalization) [7]. One aspect of this that is important for co-expression is that it renders the data compositional in a mathematical sense: each gene’s expression level is really a fraction of the total. This could be problematic for co-expression analysis because it induces unintended co-variation, particularly among low expressing genes. To investigate the functional consequences of this for co-expression analysis, we again did a similar aggregation procedure for our eight batches using either standardized (CPM) data or unstandardized (UMI) data, then used neighbor voting in cross-validation to test the connectivity of GO slim gene sets. Similar to our meta-analysis results, GO slim performance was fairly low (mean AUROC UMI = 0.54 +/– 0.002, mean AUROC CPM = 0.54 +/– 0.001) but did rise with aggregation (UMI = 0.56, CPM = 0.55) suggesting replicable functional connectivity among batches (Fig. 3a). UMI networks had higher performance both for GO slim and also for a gene set of specific functional relevance to our data, post-synaptic proteome genes (inset Fig. 3a, mean AUROC UMI = 0.73 +/– 0.004, mean AUROC CPM = 0.69 +/– 0.005, see [36] for PSD gene list).

To begin to explore the impacts of other technical factors on connectivity (outlined in Table 2), we compared the semantic similarity of the top 1 % of connections across the genes common to all networks. Semantic similarity assessment is useful in this scenario as it provides a robust, single value per network across all functions [20]. Similar to what we observed with the whole network functional connectivity assay, the top 1 % of connections in the UMI aggregate have higher semantic similarity than those of the CPM aggregate (mean UMI = 4.90 +/– 0.058, mean CPM = 3.52 +/– 0.054). As might be expected, binary aggregates, which have the least information about expression variation, showed the lowest semantic similarity among the top 1 % of connections (Fig. 3b, mean = 3.26 +/– 0.054 overlapping GO functions). By contrast, UMI networks with zeroes excluded showed semantic similarity extremely similar to UMI networks with zeroes included (mean UMI zeroes excluded = 4.97 +/– 0.058 vs. mean UMI 4.90 +/– 0.058). Because excluding zeroes will preferentially impact low-expressing genes, this suggests they play little aggregate role in the functional connectivity of the network. This is reinforced by the lower performance of the binary aggregate, where high-expressing genes will show little variation and therefore little connectivity. We focus more specifically on the role of expression in network topology and function below (see “Identifying and controlling for expression level confounds in co-expression network analysis”).

Another interesting result concerned the effects of batch correction. Though meta-analysis of co-expression across batches is one method to reduce the influence of technical factors (i.e. merging networks), in other instances, researchers may wish to correct for technical factors directly by using batch correction algorithms (i.e. merging data). To test the effects of two well-known methods, we performed batch correction for each cell type using Combat [37], an empirical Bayes method of data adjustment that requires knowledge of batches, as well as RUV [38], which uses factor analysis to estimate batch effects based on data properties and can also incorporate known control genes (in our case, ERCC spike-ins) to normalize data. Following this, batch-corrected cell-type networks were aggregated. Notably, Combat corrected UMI data showed much higher semantic similarity among top connections than RUV corrected data (Fig. 3b, mean Combat = 4.90 +/– 0.058, mean RUV = 3.72 +/– 0.055). Furthermore, comparison of the distribution of connections indicated no significant differences between Combat, UMI, or UMI excluding zeroes (*p* >0.2 for all comparisons, Kolmogorov-Smirnoff test); however, a significant difference was found between these and the distribution of connections from the batch-affected network despite having similar aggregate semantic similarity (Fig. 3b, mean batch affected semantic similarity = 4.67 +/– 0.058, *p* <1E-3 for all pairwise comparisons, Kolmogorov-Smirnov test). Indeed, all other networks exhibited distributions that were distinctive (all other pairwise comparisons *p* <1E-3, Kolmogorov-Smirnoff test).

#### Characterization with respect to topology

Next, we aimed to characterize networks in an unsupervised way, without relying on the GO to determine functional overlaps. We did this in two ways, the first of which was to threshold networks at 1 % and perform pairwise comparisons of gene-gene connections using the Jaccard similarity index (JI), where 0 indicates no overlap and 1 means perfect overlap (Fig. 3c). The results of this assessment were strongly concordant with our previous observations. As with the semantic similarity test, we found that the top 1 % of connections from UMI, UMI excluding zeroes, and Combat-corrected networks had the highest degree of overlap (JI >0.5 for all pairwise comparisons), and that the binary aggregate was the most dissimilar from all other networks (JI <0.03 for all pairwise comparisons). Interestingly, this analysis provided further insight into the effect of RUV correction using ERCC spike-ins, which had the highest similarity to the CPM aggregate (JI_RUV-CPM_ = 0.1, all others <0.06), as well as higher similarity to the UMI aggregate than the Binary and CPM networks do (JI_RUV-UMI_ = 0.06, JI_CPM-UMI_ = 0.03, JI_Binary-UMI_ = 0.01). This suggests that RUV has some positive batch-correcting effects (similar to Combat) but these are offset by the negative compositional effects caused by ERCC-dependent normalization. In addition to the aggregate assessment, we also tested the individual cell-type networks, with similar results.

For our final test, we took advantage of our experimental design to describe the replicability of network co-expression distributions, considering each batch network as a replicate. This was done by comparing the standard deviation of aggregate co-expression distributions to the distribution of a randomly permuted null aggregate (Fig. 3d). Because each of the individual networks is rank-standardized to a uniform distribution, we can determine the replicability of co-expression—whether particular gene pairs are highly ranked in each replicate network—simply by assessing the standard deviation of the connectivities in the aggregate network. Under the assumption of independence between networks, the aggregate co-expression distribution is the convolution of the (uniform) co-expression distributions of the underlying networks. Greater replicability among batches will yield a wider distribution than would be expected by chance. Note that replicability cannot be comparably assessed for the batch-corrected data because their aggregates do not treat each batch independently. To determine the relative contribution of replicability across the zero and non-zero values of the networks, we compared the distribution of aggregates built from binary data to those built from pairwise correlations excluding zeroes and from all data. As expected, binary and pairwise aggregates showed lower variation than full networks as they contain only a fraction of the signal (Fig. 3d). Furthermore, all aggregate distributions have higher standard deviations than random, indicating a greater degree of replicability than might be expected by chance (binary and pairwise variation ~1.4–1.6-fold above random, CPM 1.8-fold, and UMI 2.1-fold).

Our combination of functional and topological analyses suggests that UMI-based networks show the greatest degree of functional connectivity, semantic similarity, and replicability. Interestingly, Combat-corrected aggregates also showed a high degree of semantic similarity and topological overlap with the UMI aggregate, providing a useful and novel validation of batch correction based on co-expression analysis. RUV and CPM aggregates, on the other hand, show lower performance and semantic similarity though, notably, replicability of CPM networks is comparable to that of the UMI networks as evidenced by the topological and aggregation tests. We suggest that this is likely because sample-level standardization induces artefactual co-variation (discussed in [39]) which is replicable across batches. It is plausible that improvements in either normalization or network construction may diminish these effects. The common co-expression approach of only considering relative gene-gene correlations (e.g. top 1 %, hierarchical clustering, etc.) is likely helpful for precisely this reason. Finally, our experiments to determine the contribution of zero and non-zero values to co-expression suggest that expression level may play an important role in driving functional connectivity. We explore this in further detail below.

### Identifying and controlling for expression level confounds in co-expression network analysis

#### Expression level influences topology and functional connectivity in UMI networks

**Fig. 4:**
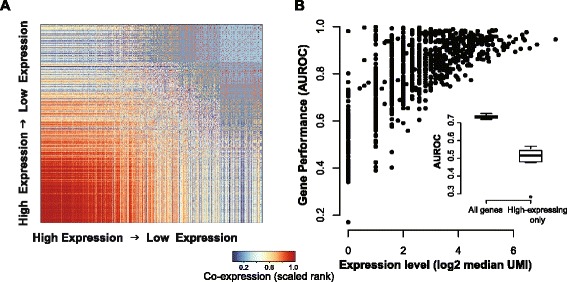
Functional connectivity is dependent on expression level in UMI networks. **a** A UMI-based network is plotted as a function of gene expression level, with co-expression values for highly expressed genes in the *bottom left corner* and lowly expressed genes in the *top right corner*. A characteristic *sunset pattern* is observed in A, with the highest co-expression values occurring between highly expressed genes. **b** AUROC for synaptic set performance in n-fold cross-validation is plotted against median expression level for each held-out gene (plot shows the results from one network). A strong positive relationship was observed between expression level and performance (mean r_s_ = 0.72 +/– 0.038). *Inset: Box-and-whisker plot* of synaptic set performance in networks made from all genes compared to networks made from high expressing genes only (median > =16 in each batch). A significant reduction in performance was observed in filtered networks (mean AUROC in all gene networks = 0.73 +/– 0.004, mean AUROC in high expressing networks = 0.49 +/– 0.03, *p* <0.0002 Wilcoxon rank sum test, n = 8)

Based on our previous observations, we sought to visualize the impact of expression level on network topology by directly plotting co-expression networks with genes ordered by the number of non-zero values they take. Co-expression is not generally shown in this way, likely because the topological structure of the data is normally too complex to admit it [40]. Strikingly, in our case, UMI network plots are highly structured, with much stronger co-expression of high expressing genes than low expressing genes (representative plot in Fig. 4a, mean correlation between node degree and median expression level = 0.77 +/– 0.04). Note that this node degree correlation is not expected with all analysis methods. Standardization practices that induce strong co-variation among lowly expressed genes will show the opposite direction of effect (see Additional file 4: Table S3 and Additional file 5: Figure S2 for more detail).

We hypothesized that the expression-level dependency within the UMI networks might explain the relative improvement of the synaptic gene set performance compared to other GO categories, reported in the previous section. We formalized this with an n-fold cross-validation exercise, where we removed labels from each gene in the synaptic set one by one and assessed performance. There was a strong relationship between gene expression and performance (Fig. 4b, mean r_s_ = 0.72 +/– 0.04), indicating that high expression is sufficient to drive results. We further demonstrated this by testing the performance of expression level-matched controls; these were very similar to the real gene set in performance (mean AUROC synaptic set = 0.73 +/– 0.004, mean AUROC 100 control sets = 0.72 ± 0.005). To determine whether high expression is necessary for high performance, we restricted networks to only include high expressing genes (with median expression > =16 counts). This yielded much smaller networks, between 51 and 1368 genes in size (median = 227 genes), of which the synaptic set made up ~30 % (mean = 29.4 % ± 1.4 %). Synaptic gene set performance was greatly reduced in these networks (inset Fig. 4b, mean 0.49 ± 0.03, *p* <0.0002 compared to non-thresholded networks, Wilcoxon rank sum test, n = 8).

These results show that UMI-based networks contain functional information; however, the primary feature is their dependence on expression level. A natural next step was to consider whether a subset of genes is biased and may be removed, yielding a high confidence network without topological dependency on expression. We tested this by applying an expression threshold to only include genes with non-zero values in at least half of the samples within each batch, yielding 1346 genes in the intersect. This is a sufficiently stringent criterion that we have removed the majority of genes (~92 %), but the sunset structure remains robustly intact (Additional file 5: Figure S2). Indeed, even batch-corrected networks display this topology (Additional file 5: Figure S2). The dependency on expression appears to be continuously distributed across all genes; if the genes most dependent are removed, the relationship is still present among the remainder. This highlights the necessity for appropriate controls, like the use of expression-matched gene sets, as opposed to filtering. This also suggests that a simpler analysis, like functional enrichment of highly expressed genes, may provide as much information as co-expression using data of this type.

#### Performance variation can be predicted by expression level and associated technical features

Individual batch networks show some variation in performance, and we wished to determine whether this might be explained by the same principles we outlined above. In keeping with our earlier result, we find a strong relationship between the average AUROC across GO functions for each batch network and the total level of expression within that batch (Additional file 6: Figure S3, r_s_ = 0.62 between AUROC and total molecule counts), potentially related to selection bias within the data (Additional file 6: Figure S3, r_s_ = 0.88 between AUROC and the number of detected genes). One important consideration is whether these differences in data quality are trivially correctable by, for example, increased sequencing depth. While our level of over-sequencing (i.e. number of reads per UMI) was in the range of previous reports using it as a QC metric [41], the correlation between over-sequencing and performance was strongly negative (Additional file 6: Figure S3, r_s_ = –0.76). While this may seem counter-intuitive at first glance, it is easier to understand when reversed—low complexity data are easier to sequence and have lower performance—but does suggest that using over-sequencing for quality control cannot be done naively. Interestingly, this relationship between over-sequencing and selection bias is only visible through the window provided by our use of replicates: the two properties have little correlation across all samples (Additional file 6: Figure S3, r_s_ = –0.02).

#### Expression level explains the node degree dependency of functional connectivity in the single-cell aggregate network

Having observed a strong dependency of network connectivity on expression level in our UMI networks, we sought to determine whether this could be observed in other single-cell networks. The distribution of correlations varied widely across networks, concordant with the fact that data derive from many experiments and was subject to a range of normalization procedures (Additional file 7: Figure S4, –0.53 < r_s_ < 0.85). Because the networks are very large, this range of correlation values is enormously more variable (and positive) than expected by chance with mean absolute z-scores of 28.4 after Fisher’s transformation. While the exact relationship with network topology will vary depending on normalization, expression level is critical to consider in virtually every case. Interestingly the data that were most similar to ours, a cortical interneuron network based on UMI data, also showed a similarly high positive correlation between node degree and median expression (r_s_ = 0.71).

**Fig. 5:**
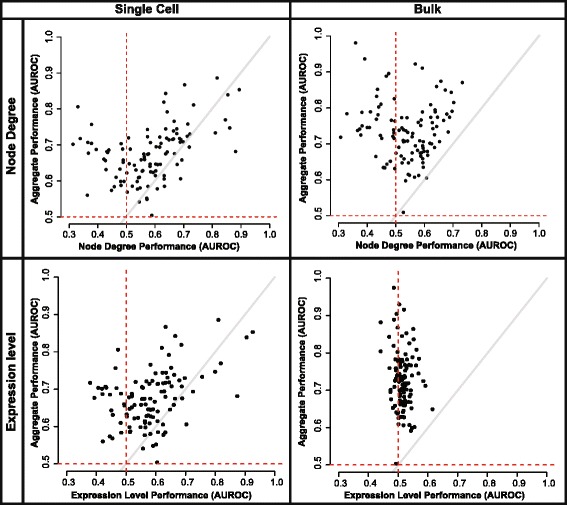
Node degree and network performance are predicted by expression level in the single-cell aggregate network. *Top:* GO slim AUROCs and predicted AUROCs based on node degree are plotted for single-cell and bulk aggregates (163 networks in each). Functional connectivity in both aggregates is dependent on node degree. *Bottom:* GO slim AUROCs and predicted AUROCs based on median gene expression are plotted. Single-cell aggregate performance is predicted by expression; however, there is no relationship between expression and bulk aggregate performance

Our meta-analytic aggregate networks have higher performance than any individual network, so to trace the functional impact of expression level more broadly than the synaptic gene set we focused on using the aggregate networks. Previous work from our lab has shown that functional connectivity in gene networks can be predicted from the node degree of functional genes, with high node degree genes being good candidates for many functions [24]. We assess this by using the node degree as a predictor for each gene function; we control for the role of node degree by making predictions using it alone (“Node degree performance”) and determining how much of a given GO group’s performance within the network could be attributable to this factor. Both the bulk and single-cell aggregate network performance showed a characteristic V-shaped dependency on node degree due to our use of signed networks (Fig. 5, top right panel). Within the scRNA-seq co-expression networks we can, again, trace this back to a dependency on expression level (Fig. 5, bottom left panel). However, this is not the case for the bulk RNA-seq aggregate (Fig. 5, bottom right panel), possibly because its higher performance means it is powered sufficiently to overcome single-study (or even single pipeline) technical artifacts and is therefore robust to weak expression level variation. Generalizing from this, because individual bulk studies are not as well powered as the aggregate networks, we might hypothesize that where performance variation exists, it may again derive from simple data features such as expression level. We tested this through a re-analysis of the BrainSpan data, where both expression level variation and functional specificity in co-expression have been previously identified.

#### Expression level predicts autism candidate gene connectivity in BrainSpan networks

Our meta-analysis results indicate that aggregation of bulk RNA-seq data generates a network with robust, functionally convergent performance that is not dependent on expression level. However, this does not preclude the notion that expression level differences can explain variation in performance where it does occur. For example, one of the most exciting co-expression results in recent years has been the finding that candidate genes for neuropsychiatric disorders such as autism spectrum disorder (ASD) and schizophrenia tend to be co-expressed in the brain [3, 42] and vary in their degree of co-expression by developmental stage [25, 43]. Also interesting are the independent reports of ASD gene differential expression between pre-natal and post-natal data within BrainSpan [44]. Our results suggest an obvious link between these results and also the possibility that the reported differential connectivity may be explained by simpler data features.

**Fig. 6:**
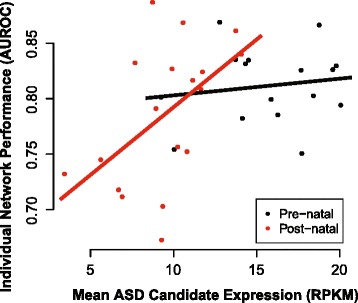
Differential connectivity of autism candidate genes in BrainSpan networks is predicted by expression level. Networks were built using BrainSpan RNA-seq data for individuals with ten or more samples (16 pre-natal, 18 post-natal networks). Connectivity of autism candidate genes is plotted against mean expression, with linear regression lines for pre-natal and post-natal networks colored in *black* and *red* (adjusted R^2^ = –0.05, = 0.23, respectively)

To test this, we generated networks for every individual in BrainSpan where ten or more brain region samples were available (Additional file 8: Table S4), then assessed the connectivity of ASD candidate genes (see [45] for gene list) using the same general machine learning framework we used to measure the connectivity of GO groups and synaptic genes in previous sections. While this method is quite different from the more customized analyses underlying initial reports, we were able to replicate the main prior claims of differential connectivity (Fig. 6). We observed modestly, but consistently, higher performance in pre-natal networks and more variable performance in post-natal networks (pre-natal AUROC = 0.81 +/– 0.009, post-natal AUROC = 0.79 +/– 0.015). Encouragingly, it was principally the co-expression performance within the post-natal networks which showed a significant association with the expression level of the ASD genes (adjusted R^2^ = 0.23, *p* <0.05). That is, where the differences in performance are less likely to reflect relevance to disease, we found greater relevance for an artefactual origin. There was no association between age and co-expression performance within the post-natal data (Additional file 9: Figure S5), indicating the expression level is likely useful as a fundamental control in itself. While some of the difference between pre-natal and post-natal co-expression may be due to differences in expression level, the variation within the pre-natal data itself showed no such trend, so that comparisons within pre-natal data may reflect co-variation not explained by expression level alone.

## Conclusions

Co-expression analyses comprise a diverse and complex set of methods that have, historically, found widest application in otherwise hard-to-advance areas, such as predicting novel gene functions (e.g. [46, 47]) or characterizing disease genes (e.g. [48, 49]). The number of co-expression methods that have been developed is large since it builds on expression analysis to include network construction [50] as well as the interpretation of that network. Amid this diversity, there are community practices which have evolved to make co-expression analysis robust, but whose influence is rarely formally assessed (although see [51]). Given this mixture of explicit and implicit dependencies, interpreting the impact of novel technical variation in scRNA-seq on co-expression is challenging: how can we assess a wide enough corpus of methods to determine which are working?

Our approach has been to employ comparatively simple methods whose downstream effects in the data are likely to generalize. Thus, our claim is not that correlation and pairwise correlation excluding zeroes (for example) are optimal methods for computing gene-gene similarity, but that novel methods with data-specific dependencies will likely show downstream impacts that are some combination of the simple, easily explained, and quite different, effects we observe. As the first step in motivating this approach, we conducted a wide-ranging meta-analysis of single-cell and bulk expression data, re-analyzing it and testing for aggregate functionality in the derived networks. The steady rise in performance with quantity of data suggests our methods for assessment are robust to practices within the field and allowed us to investigate the role of possible sources of co-expression within the data. Indeed, the use of a more sophisticated machine-learning algorithm, GeneMANIA, provided comparable results. Having established that our assessment methods were likely to generalize, we designed an experiment to elucidate replicability within single-cell co-expression, including factors which cannot be easily assessed in meta-analysis. We sampled across a non-trivial class of cellular variation in batches, each of which could be used to assess co-expression performance and replicability. These results provide some general guidelines for interpreting co-expression in single-cell data, and particularly the importance of variation in expression level. Finally, we validate the relevance of derived heuristics by applying them in the complex and important characterization of autism co-expression within the brain.

Our analysis suggests four concrete recommendations targeted to co-expression:

1. Ideally experiments should be run in replicate and then the separate replicate networks should be aggregated. Our extensive analysis of bulk and single-cell co-expression provides evidence that any individual experiment has low predictive power, regardless of sample size, but that aggregation improves performance. This also allows researchers to estimate technical and biological variation in a meaningful way and to define patterns that are robust. Replication has additional benefits for single-cell experiments which seek to delineate cell types or cell sub-types: finding similar proportions of cells across replicates provides an estimate of clustering accuracy (e.g. as in [7]).
2. Baseline functional connectivity, measured against GO slim, should be reported using neighbor voting or publicly available function prediction algorithms, such as GeneMANIA [28]. This will provide researchers with a benchmark for expected performance and allow for cross-laboratory comparisons.
3. Performance should be evaluated with respect to possible technical effects. In supervised methods, this may take the form of plotting leave-one-out performance versus the putative confound. In unsupervised methods, the degree to which the technical effect is confounded with modules should be reported via ANOVA. Particular factors to consider are expression level and number of drop-outs. Plotting networks with respect to potential confounds may also be helpful for exploratory analysis (as in Fig. 4a and Additional file 5: Figure S2).
4. Co-expression specificity should be checked against our meta-analytic aggregates. Alongside this paper we have provided the top 0.5 % of connections in the bulk and single-cell aggregate networks, as well as the top 0.5 % of connections in each of the underlying single-cell networks. Genes co-expressed in a cell type- or condition-specific manner should not be present in the bulk or single-cell aggregates. Additional evidence for condition specificity may be derived from comparison to comparable single cell type networks and should be reported.

One aspect of single-cell analysis that has yet to be resolved is how best to normalize data. As we and others have shown, normalization is not trivial for scRNA-seq, likely due to the current state of the technology, which samples inconsistently from the RNA pool within cells. Even a relatively conservative rank standardization will induce strong artefactual correlations among low expressing genes because different numbers of genes are expressed in each sample and across batches. Likewise, batch correction is powerful but potentially dangerous, and must be applied carefully or researchers risk the removal of interesting variation [52]. While removal of low expressing genes may be possible in some cases, this will make comparison between studies confounded by selection bias. These sorts of basic biases seem also likely to confound even sophisticated statistical techniques until they are much better characterized within the data. To solve this problem, we suggest keeping all of the data and implementing meta-analytic methods which explicitly assess replicability as this is more likely to provide a general solution.

Indeed, as suggested by the BrainSpan analysis, many of the heuristics we derive for single-cell data apply to bulk data as well, where their presentation may be less immediately obvious. Notably, expression level explained variation in ASD gene co-expression performance only in the post-natal data, where the ASD gene expression was comparatively low to begin with, and so it is likelier for that variation to make sense as a confound. Thus, our results are encouraging for the significance of the BrainSpan autism analyses in suggesting that the pre-natal performance dependencies, interpreted as providing support for the neurodevelopmental features of autism, are not the result of expression level confounds. More generally, we suggest that the particular prominence of technical variance with single-cell data [29] makes it a useful resource for determining the downstream impact of potential artefacts. For this reason, future assessment of replicability within co-expression would likely benefit from a focus on single-cell data.

The importance of follow-up work targeting the genesis of co-expression, and using single-cell data to sample within data of constrained variability, is highlighted by the implications of our model and meta-analysis. Using GO as a reference suggests that compositional and cell-state variation exhibit very similar properties, one being redundant in the presence of the other, and using either results in network performances that are highly similar. Of course, this may reflect the use of GO which, as a “tool for the unification of biology” [53] may be less useful at dividing up biology by cell type. Alternatively, this finding is consistent with the notion that cell sub-type identity is as prominent as cell-type identity, and thus swamps cell-state co-expression. Further work to explore these hypotheses is warranted.

In addition to our topological assessments focusing on specific connectivity overlaps, module detection [51] and its replicability and condition-dependence suggest a route forward for the field. If co-expression is to be used as a means of interpreting disease gene convergence and condition specificity, the genesis of those properties are crucial to pin down. The careful exploitation of single-cell data, particularly in meta-analysis, offers a unique window into how genes work together to produce function and on what factors it depends.

## Methods

### Meta-analysis of single-cell and bulk data

We obtained 239 bulk mouse RNA-seq experiments and eight single-cell mouse RNA-seq experiments from the Gemma database [54] that were processed using RSEM [55] (version 1.2.5, and mouse reference mm10_ensembl_72). Processed data files for all other scRNA-seq experiments were downloaded directly from GEO. Cell types were defined using labels provided by authors. Where groups had profiled the same cell type in multiple batches, networks were generated for each batch. Twenty-four “cell type” networks were derived from whole tissues and could therefore contain compositional variation; however, results were robust to the removal of these experiments. Samples that were not explicitly labeled as single cells and those with fewer than 1000 genes with expression >0 were removed, as well as any cell types that were represented by fewer than 10 samples. Similarly, we restricted our bulk RNA-seq analyses to experiments with at least ten samples. Fifty-two bulk experiments (~22 %) used purified cell populations, which, though they are not compositional in the sense of having multiple cell types represented, are preferentially non-state by dint of averaging across cell-state variation within each sample.

Data analysis was performed in R using custom scripts [56]. Only genes appearing on both Affymetrix GeneChip Mouse Gene 2.0 ST array (902119) and the UCSC known gene list were considered. The mean value was taken for all genes with more than one expression value assigned. Networks were built from Spearman correlations and undefined correlations were set to zero. Edge weight was defined as the rank of the correlation coefficient within the network and node degree was calculated as the summation of all the weights connected to a given node [24]. Aggregation was performed by averaging ranked correlation coefficients across networks, then re-ranking and standardizing values between 0 and 1. We obtained gene annotations from the GO Consortium “goslim_generic” (August 2015). These were filtered for terms appearing in the GO Consortium mouse annotations “gene_association.mgi.gz” (December 2014) and for gene sets with between 20 and 1000 genes, leaving 108 GO groups with 9421 associated genes.

Functional connectivity was measured using a neighbor-voting algorithm, in which genes are scored based on the fraction of the genes to which they are connected which possess a given property [21, 26, 57]. The “performance” at this task is defined as the AUROC after threefold cross-validation [24] using GO groups, i.e. how well the network connectivity allows the reconstruction of known gene functions. Networks are scored by the average of the AUROCs across GO functions.

### Animals and manual cell sorting

*Nkx2.1*-CreER mice [58] and *Pv*-ires-Cre [59] animals were bred separately to Ai14 reporter [60] to label ChC and Pv basket cells in the cortex. ChCs were enriched in frontal cortex with tamoxifen induction at E17.5 [58]. Mice were bred and maintained according to animal husbandry protocols at Cold Spring Harbor Laboratory (Institutional Animal Care and Use Committee reference number 16-13-09-8) with access to food and water *ad libitum* and 12 h light-dark cycle. Adult animals (P28-35) were sacrificed by cervical dislocation to harvest brains for single-cell sorting.

Single cells were collected by manual sorting procedure as detailed in Sugino et al. [61]. Brains were sectioned to 300 μm using a cooled stage vibratome with circulating oxygenated artificial cerebrospinal fluid. Sections were blocked in AP5, CNQX, and TTX cocktail to prevent excitotoxic cell death and then treated with mild protease. Brain regions of interest were microdissected and triturated to dissociate the cells. Dissociated cells were put into in a Petri dish and RFP-positive cells were collected into single patch pipette capillaries and dispensed into single tubes containing RNAseOUT (Invitrogen), ERCC spike-in RNAs in 1:400 K dilution, sample specific RT primers for a total of 1 μL volume. Collected cells were flash frozen in liquid nitrogen and stored at –80 °C until processed. Individual sample details can be found in Additional file 3: Table S2.

### RNA amplification, Illumina library prep, and sequencing

RNA was linearly amplified using two rounds of in vitro transcription using MessageAmp-II kit (Life Technologies) according to the manufacturer’s recommended protocol. Amplified aRNA was reverse transcribed using SuperScript-III enzyme (Invitrogen) and made into cDNA library using Illumina TruSeq small RNA library preparation kit using 7–11 cycles of PCR according to the manufacturer’s protocol. The resulting library was size-selected using SPRISelect magnetic beads (Agencourt) and paired-end sequenced for 101 bp in Illumina HiSeq.

### Mapping and QC

Bowtie (v 0.12.7) was used for sequence alignment of polyA primed reads to the mouse reference genome (mm9), while read1 sequences were used for varietal tag (a.k.a. UMI) counting. Using a custom python script, multiple reads to the same gene with the same tag sequences were rejected and only counted as one, such that only mapped sequences with unique tags were retained and tallied for each mRNA for each cell. Two cells failed to amplify and resulted in 0 expressed genes. These were removed prior to further analysis. For the remainder of the dataset, the mean number of genes detected with >0 counts is 5407.5 ± 189.8 per cell, and the average level of over-sequencing across all genes is 4.4-fold which is in line with previous single-cell studies [41, 62, 63].

### Replicability analyses

For replicability analyses, networks were built for individual batches then aggregated as described above. Details about network generation can also be found in Table 2. As stated in the results, there were three aspects of the data that we wished to explore for their contribution to network replicability; namely, the role of zeroes, expression level, and normalization. To clarify the role of zeroes, zeroes were set to NA then networks were built by taking the pairwise Spearman correlation across genes using either counts or CPM values. To test the importance of variation in non-zero expression level, “binary” networks were built by setting all non-zero values to one prior to generating networks. To test the effect of normalization in addition to CPM, batch-corrected aggregates were built by summating ChC and Pv networks where batch correction had been performed within each cell type using either Combat as implemented in the sva package (v 3.14.0), or using the RUVg function of the RUV package (v 1.2.0) with ERCC spike-ins as control genes. Only genes that were non-zero in more than 50 % of both ChC and Pv cells were included (3642 genes total). The “batch-affected” network was built by taking Spearman correlations across all 126 samples. For UMI and CPM networks, functional connectivity and connectivity of synaptic genes (downloaded from the Genes to Cognition database [36]) were assessed as described above.

Semantic similarity and pairwise topological overlap analyses were performed on networks filtered to have the same set of genes as those in the batch-corrected networks. For the semantic similarity test, a gene-gene matrix was generated counting the number of times each gene pair had the same GO function, using only non-IEA functions with 20 to 1000 genes. The top 1 % of each network was compared to this, and the mean number of common GO groups among the top 1 % was plotted. Topological overlap was measured by direct pairwise comparisons of gene-gene connections within the top 1 % of networks using the JI. Replicable topological overlap within conditions was assessed for networks containing all genes. For this we reported the standard deviation of the aggregate co-expression values, using the aggregation of eight randomly generated rank-standardized networks as the null.

### Testing expression level dependence

Spearman correlations between node degree and median expression were performed for all networks, excluding any genes with undefined correlations to all other genes (i.e. node degree = 1). For our data, neighbor voting in combination with n-fold cross-validation was used to evaluate the influence of expression level on performance. Second, synaptic set performance was compared to the performance of 100 randomly chosen gene sets with similar expression levels. Expression-matched sets were generated by binning the synaptic set genes into quartiles and choosing the same number of genes from each quartile. Finally, synaptic set performance was compared between networks containing all genes and networks that were stringently filtered to include only those genes with median expression >16 UMIs. For the meta-analysis, we tested the dependence of aggregate network performance on both node degree and median expression by generating prediction vectors for both factors and calculating the AUROC analytically:

$$ AU{C}_j=1-\left({\displaystyle \sum_{i\Big|Gen{e}_{i\epsilon }G{O}_j}} Rank{s}_i-\frac{N_{Pos}\ast \left({N}_{Pos}+1\right)}{2}/\left({N}_{Pos}*{N}_{Neg}\right)\right) $$

where “Ranks” are the ranks of the hidden positives, N_pos_ is the number of true positives, and N_Neg_ is the number of true negatives.

BrainSpan RNA-seq data were downloaded from the BrainSpan consortium on July 2015 [64, 65]. Networks were generated as described above for all individuals with ten or more samples available (listed in Additional file 8: Table S4). Connectivity of ASD genes was assessed with the neighbor-voting algorithm and AUROC scores were regressed against mean ASD gene RPKM values for each individual.

### Availability of supporting data

A Github repository containing R scripts and parsed data can be found online [56]. Raw data files, parsed data, and metadata have been uploaded to GEO (accession GSE75049). Aggregate networks and individual single-cell networks are available to download and are linked from our Github page.

### Ethics

Mice were bred and maintained according to animal husbandry protocols at Cold Spring Harbor Laboratory (Institutional Animal Care and Use Committee reference number 16-13-09-8).

## Box 1. Glossary of terms

Co-expression network – A representation of gene-gene relationships, built by measuring expression profile similarity across samples. Genes are denoted as nodes in the network and the connections between genes are called edges. A weighted network contains information about the strength of the connections between genes. Signed networks contain information about the direction of the association (i.e. positive vs. negative correlations).

Fully connected network – A network containing a connection between every gene-gene pair.

Sparse or thresholded network – A network containing information only about gene pairs with strong connections. To generate sparse networks, a threshold will be picked to define strong connections; the top 0.5 % of the network (i.e. standardized edge weights > =0.995) is commonly used [20].

Node degree – A measure of gene (a.k.a. node) connectivity within a network. A gene’s node degree is calculated by adding up the strength of its connections to other genes (a.k.a. edge weights). Genes with high node degree are commonly referred to as hub genes.

Functional connectivity – Refers to gene-gene connections that overlap with known cellular or biological processes as defined by the Gene Ontology (i.e. gene functions).

Neighbor voting – A method to classify genes into known categories based on gene-gene connections (i.e. the gene’s neighborhood). In our study, candidate genes were scored by dividing the sum of the ranks of the gene connections (i.e. edge weights) within the training set by the sum of the ranks of all gene connections.

Cross-validation – A method to estimate how well the results of an analysis will generalize. The main purpose of cross-validation is to avoid overfitting to a particular dataset. There are many ways to implement cross-validation; in this study we use both threefold and n-fold cross-validation. In threefold cross-validation, we hide one-third of the known gene labels, then make predictions about unlabeled genes. This is repeated three times (i.e. for each fold). In n-fold cross-validation, we hide labels one at a time, making predictions as each label is hidden, until we have tested all of the genes within a particular gene set.

Performance – The metric used to quantify functional connectivity in our study. This refers to the area under the receiver operating characteristic curve (AUROC).

Semantic similarity – A measure of gene-gene relatedness based on the number of shared GO functions.

Topology – The layout, or connectivity patterns, of a network.

## Additional files

Additional file 1: Table S1. Bulk RNA-seq expression studies used for meta-analysis, sorted by GEO ID (GEO ID = Gene Expression Omnibus Identifier). The table contains quality control and source information for each bulk RNA-seq dataset retrieved from the Gemma database. PMID = PubMed Identifier.(XLSX 72 kb) Additional file 2: Figure S1. Variability in GO group performance across networks. (PDF 1709 kb) Additional file 3: Table S2. Sample metadata for ChC and Pv single cell RNA-seq analysis. (XLSX 16 kb) Additional file 4: Table S3. Correlation between node degree and median expression for model co-expression networks with varying input. (XLSX 8 kb) Additional file 5: Figure S2. Visualizing network topology can help to reveal dependencies. (PDF 66043 kb) Additional file 6: Figure S3. Co-expression performance and sequencing depth. (PDF 670 kb) Additional file 7: Figure S4. Expression level dependency is variable in individual single cell networks. (PDF 475 kb) Additional file 8: Table S4. BrainSpan sample metadata and co-expression summary statistics. For our re-analysis of the BrainSpan data we generated co-expression networks for each individual with 10 or more samples. The table lists the sample ID, sex, age, the number of samples included in the network, and then provides summary statistics from the co-expression analysis including the AUROC of the autism candidate (ASD) genes, the mean expression of the ASD genes, and the mean performance of the network compared to GO slim. (XLSX 11 kb) Additional file 9: Figure S5. ASD co-expression performance variation is not explained by age. (PDF 534 kb)

## Abbreviations

ASD: Autism Spectrum Disorder
AUROC: Area under the receiver operating characteristic curve
ChC: Chandelier cell
CPM: Counts per million
ERCC: External RNA Controls Consortium
GBA: Guilt by association
GEO: Gene Expression Omnibus
GO: Gene Ontology
Pv: Parvalbumin
RUV: Remove unwanted variation
scRNA-seq: Single cell RNA sequencing
UMI: Unique molecular identifier
