# Supplementary material for: Exploiting single-cell expression to characterize co-expression replicability
Source: Genome Biol. 2016 May 6;17:101. doi: 10.1186/s13059-016-0964-6 (PMC4862082; doi:10.1186/s13059-016-0964-6)
Supplement: Additional file 2: Figure S1. — Variability in GO group performance across networks. (PDF 1709 kb) [file 13059_2016_964_MOESM2_ESM.pdf]

Additional file 2: Figure S1

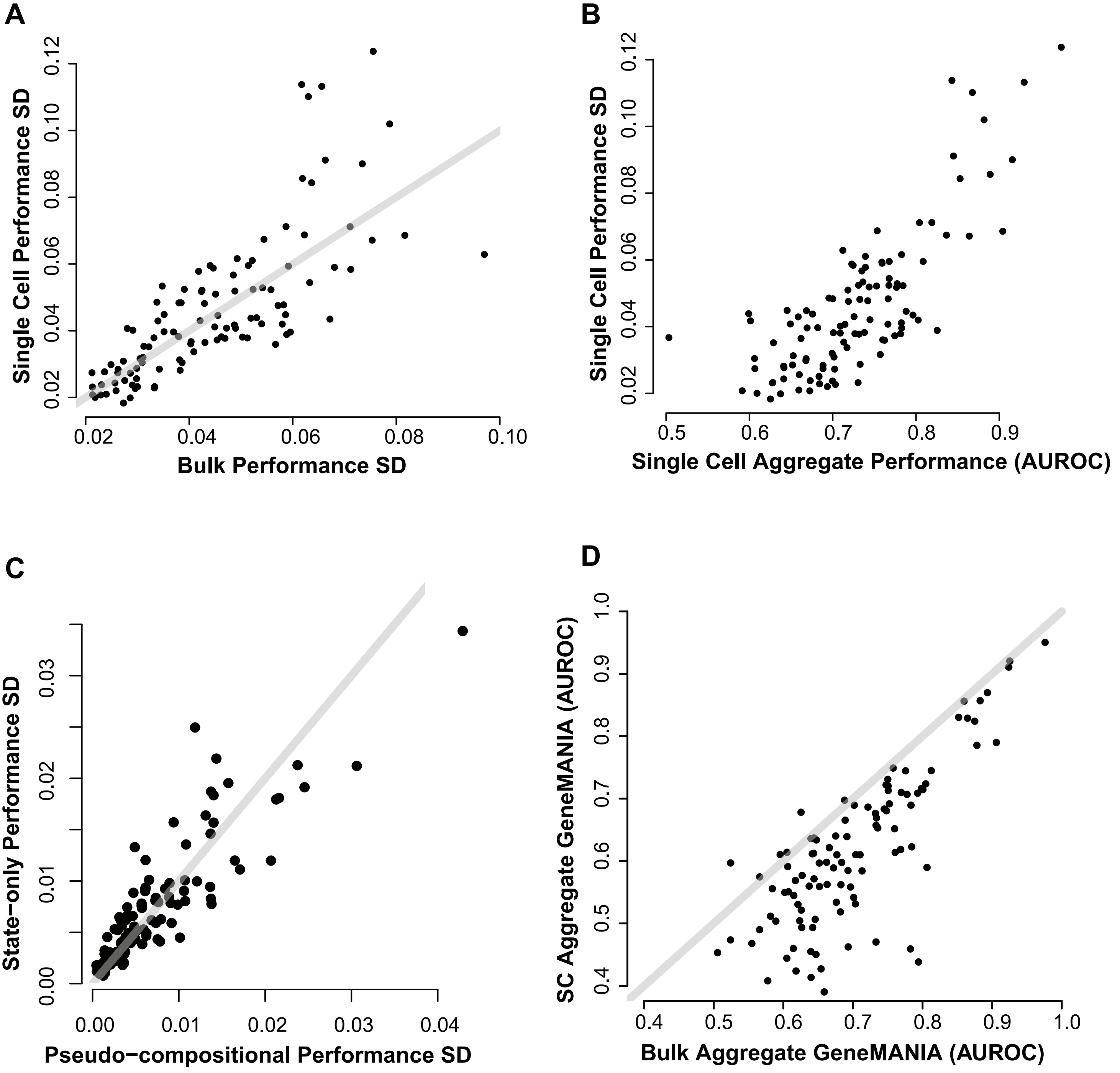

Variability in GO group performance across networks

A – Variation in performance for each GO group is compared between bulk and single cell data. The identity line is plotted in grey. Variation in performance is comparable between both data types. **B -** Standard deviation in performance across single cell networks is plotted against aggregate performance, with a clear positive trend. **C** - Standard deviation in average performance for each GO group is compared between state-only and pseudo-compositional single cell data. The identity line is plotted in grey. Variation in performance is comparable between both data types. **D -** GO slim performance calculated by geneMANIA is shown for single cell and bulk aggregates. The identity line is plotted in grey. Similar to neighbor voting, performance is correlated between both data types but single cell performance is consistently lower.
