# Supplementary material for: Exploiting single-cell expression to characterize co-expression replicability
Source: Genome Biol. 2016 May 6;17:101. doi: 10.1186/s13059-016-0964-6 (PMC4862082; doi:10.1186/s13059-016-0964-6)
Supplement: Additional file 5: Figure S2. — Visualizing network topology can help to reveal dependencies. (PDF 66043 kb) [file 13059_2016_964_MOESM5_ESM.pdf]

## Additional file 5: Figure S2

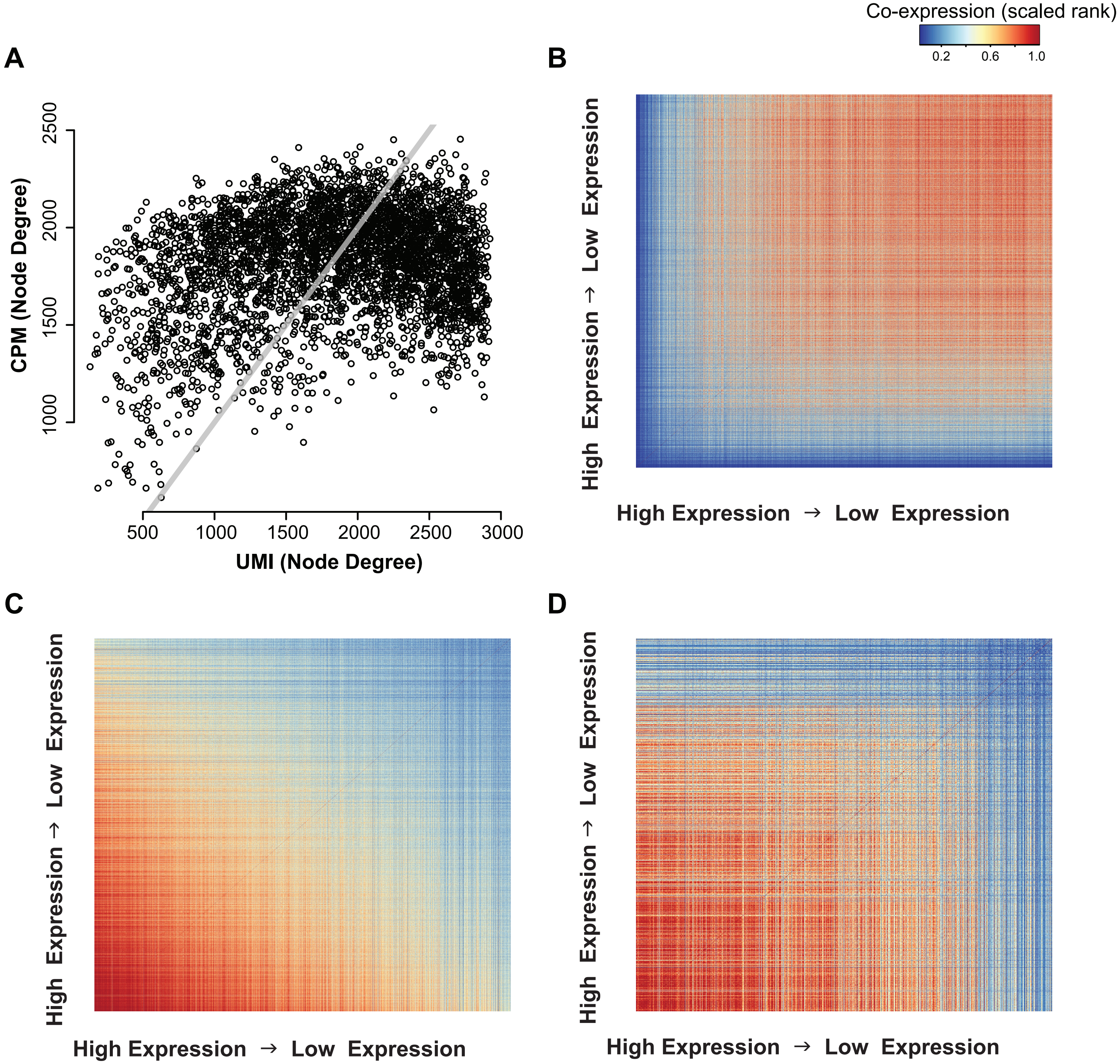


**Visualizing network topology can help to reveal dependencies**

**A** - Gene node degrees in the UMI and CPM aggregate networks are plotted. The identity line is shown in grey. While we focus on the positive relationship between node degree and expression level in UMI networks, it is important to note that standardization practices that induce strong co-variation among lowly expressed genes will show the opposite direction of effect. CPM standardization (which affects only non-zero values), produces gene node degrees which are broadly correlated with UMI for low-expressing genes, and broadly negatively correlated with UMI for high-expressing genes. **B -** "Binary" network. The co-expression in this network is driven by genes with variable on/off expression. This plot also shows that very few genes are detected in every cell (<10% of the network is dark blue). **C** - The aggregate network built from Combat-corrected ChC and Pv networks is plotted (3672 genes), showing that batch correction does not remove expression level dependency. **D** - This network was built using only a subset of genes with non-zero expression in >50% cells in all batches (1346 genes). Similar to Figure 4A, even with a limited set of genes, UMI networks maintain their characteristic "sunset" structure, indicating that filtering does not remove topological dependencies on expression level.
