# Supplementary material for: Exploiting single-cell expression to characterize co-expression replicability
Source: Genome Biol. 2016 May 6;17:101. doi: 10.1186/s13059-016-0964-6 (PMC4862082; doi:10.1186/s13059-016-0964-6)
Supplement: Additional file 6: Figure S3. — Co-expression performance and sequencing depth. (PDF 670 kb) [file 13059_2016_964_MOESM6_ESM.pdf]

Additional file 6: Figure S3

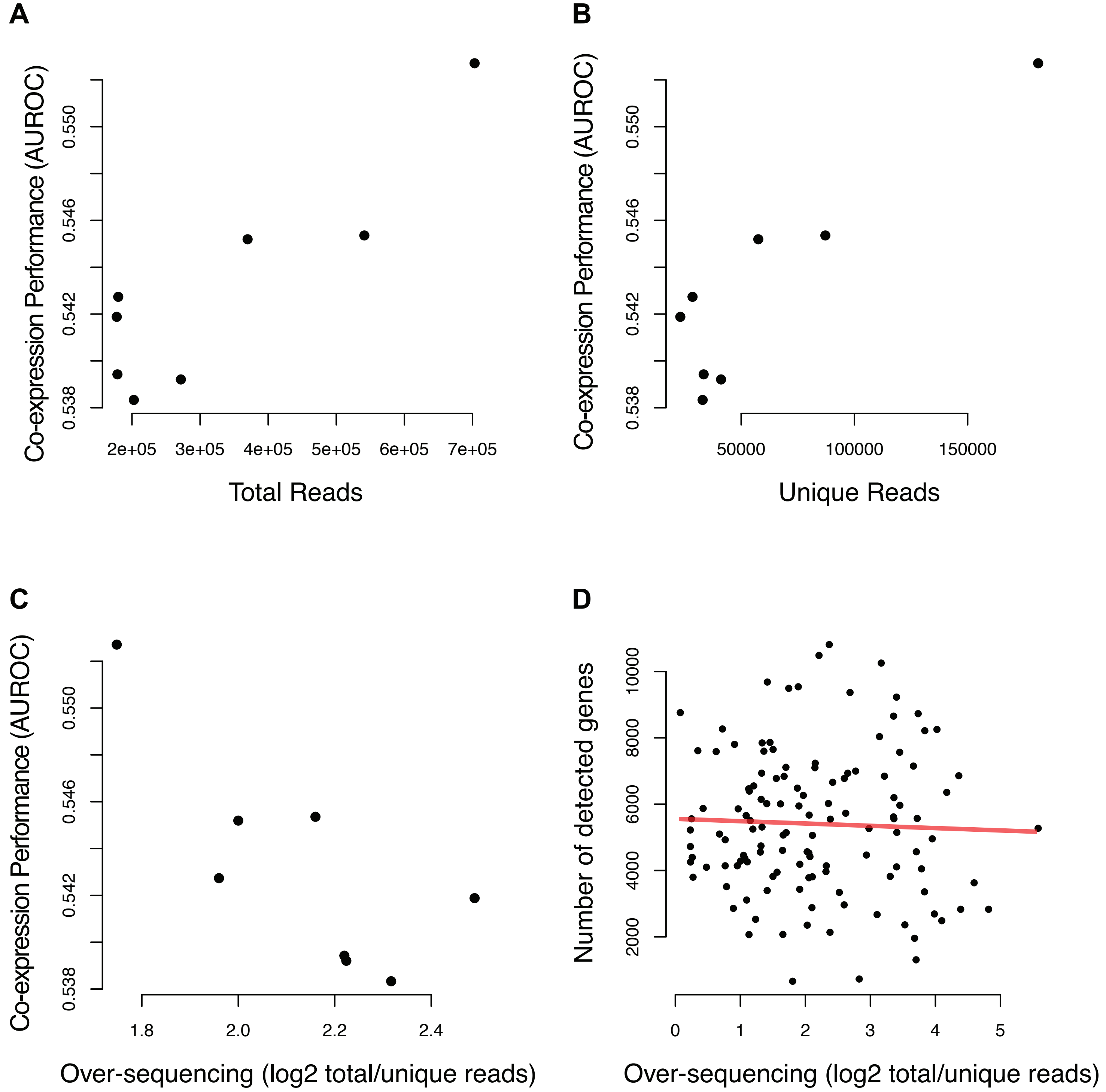

Co-expression performance and sequencing depth.
A - Co-expression performance for GO slim is plotted against the average number of molecule counts for each batch, indicating a positive relationship (r=0.62). **B** - Co-expression performance for GO slim is plotted against the average unique molecule counts for each batch, indicating a positive relationship (r=0.88) and suggesting that the association between total reads and performance may be due to the number of detected genes. **C** - Co-expression performance for GO slim in each batch is plotted against over-sequencing level, indicating a negative relationship (r=-0.76). **D** - For each sample, the number of detected genes is plotted against over-sequencing level. No relationship is discernable when all samples are considered together (r=-0.02).
