# Supplementary material for: Exploiting single-cell expression to characterize co-expression replicability
Source: Genome Biol. 2016 May 6;17:101. doi: 10.1186/s13059-016-0964-6 (PMC4862082; doi:10.1186/s13059-016-0964-6)
Supplement: Additional file 7: Figure S4. — Expression level dependency is variable in individual single cell networks. (PDF 475 kb) [file 13059_2016_964_MOESM7_ESM.pdf]

Additional file 7: Figure S4

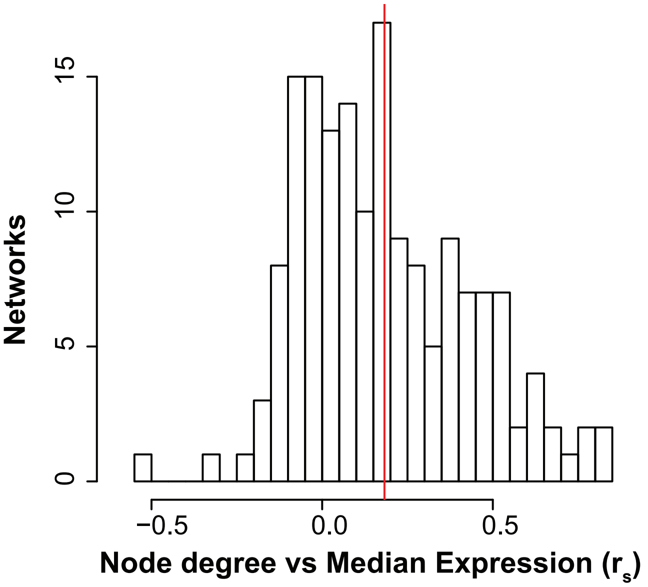

Expression level dependency is variable in individual single cell networks
For each single cell network, the correlation between node degree and median expression was calculated across every detected gene. The histogram displays the distribution of correlations across all networks, with the red line indicating the mean value (0.18). The range is large but skew-positive, suggesting some expression level dependency may be a data feature to beware of in single cell RNA-seq co-expression analysis. Also notable are the extreme negative correlations, which suggest over-correction of data.
