# Supplementary material for: Exploiting single-cell expression to characterize co-expression replicability
Source: Genome Biol. 2016 May 6;17:101. doi: 10.1186/s13059-016-0964-6 (PMC4862082; doi:10.1186/s13059-016-0964-6)
Supplement: Additional file 9: Figure S5. — ASD co-expression performance variation is not explained by age. (PDF 534 kb) [file 13059_2016_964_MOESM9_ESM.pdf]

Additional file 9: Figure S5

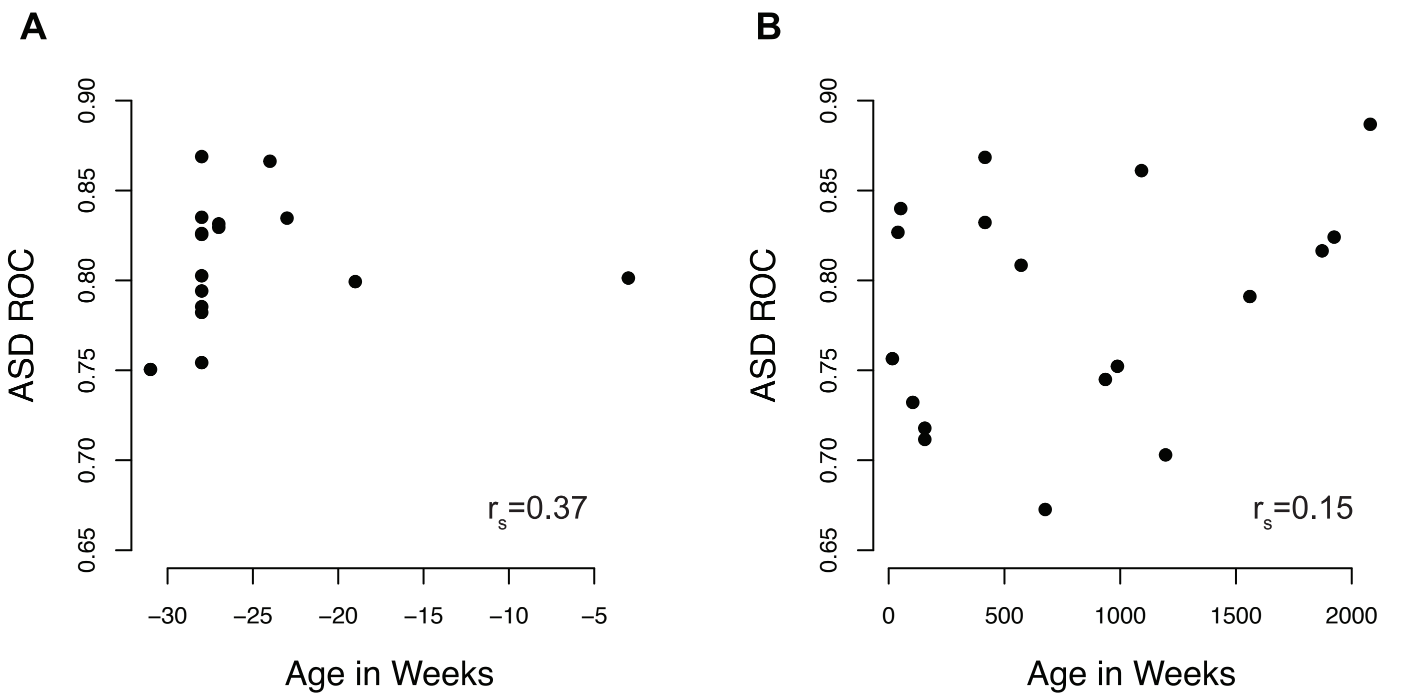

ASD co-expression performance variation is not explained by age.
A & B – Performance for ASD candidates is plotted against age in weeks for each sample (prenatal in A and postnatal in B where prenatal is calculated as 42 weeks - age). Unlike the association we observed between expression and performance, no strong association is observed with age, indicating that gene expression level can serve as an important and independent explanatory variable.
